# Supplementary material for: Green synthesis of olefin-linked covalent organic frameworks for hydrogen fuel cell applications
Source: Nat Commun. 2021 Mar 31;12:1982. doi: 10.1038/s41467-021-22288-9 (PMC8012354; doi:10.1038/s41467-021-22288-9)
Supplement: Supplementary file 1 — Supplementary Information [file 41467_2021_22288_MOESM1_ESM.pdf]

Supplementary Information

Green synthesis of olefin-linked covalent organic frameworks for hydrogen fuel cell applications

Wang, Z. *et al.*

Structure simulations:

Structural modeling of NKCOF-10 was generated using the Accelrys Materials Studio software package. The lattice model was geometry optimized using the Forcite module. Pawley refinement was applied to define the lattice parameters.

Experiment section

Synthesis of model compound 2,5-distyrylpyrazine.

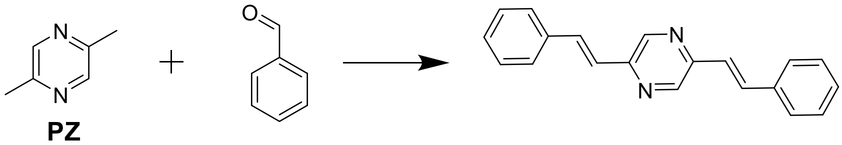

PZ

Model compound 2,5-distyrylpyrazine was prepared according to the literature.[1] A mixture of 2,5-dimethylpyrazine, benzaldehyde, and benzoic anhydride was refluxed overnight, then cooled to room temperature. The products were washed with methanol and diethyl ether to give the pure product as a yellow powder. ^{1}H NMR (400 MHz, $CDCl_3$) δ 8.44 (s, 2H), 7.58 (d, *J* = 16.0 Hz, 2H), 7.45 (d, *J* = 7.4 Hz, 4H), 7.24 (t, *J* = 7.1 Hz, 4H), 7.18 (d, *J* = 6.7 Hz, 2H), 7.02 (d, *J* = 15.8 Hz, 2H). ^{13}C NMR (101 MHz, $CDCl_3$) δ 149.10, 143.28, 136.27, 134.41, 128.86, 127.30, 124.08.

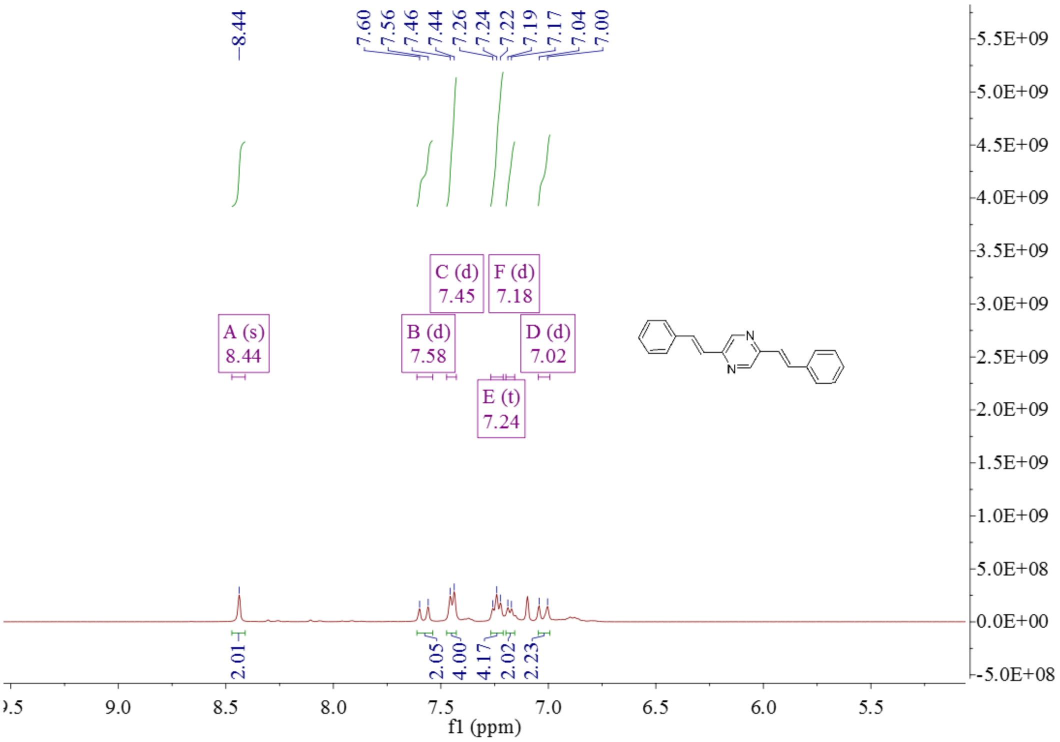

Supplementary Figure 1. ^{1}H NMR spectrum of model molecule 2,5-distyrylpyrazine.

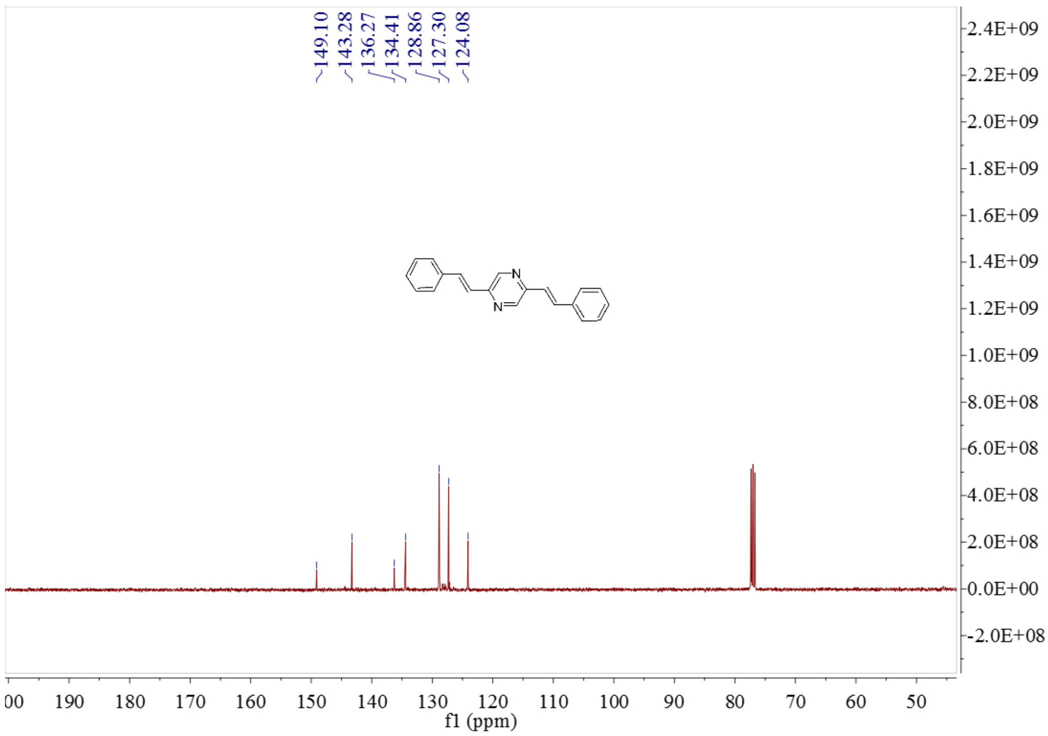

Supplementary Figure 2. ^{13}C NMR spectrum of model molecule 2,5-distyrylpyrazine.

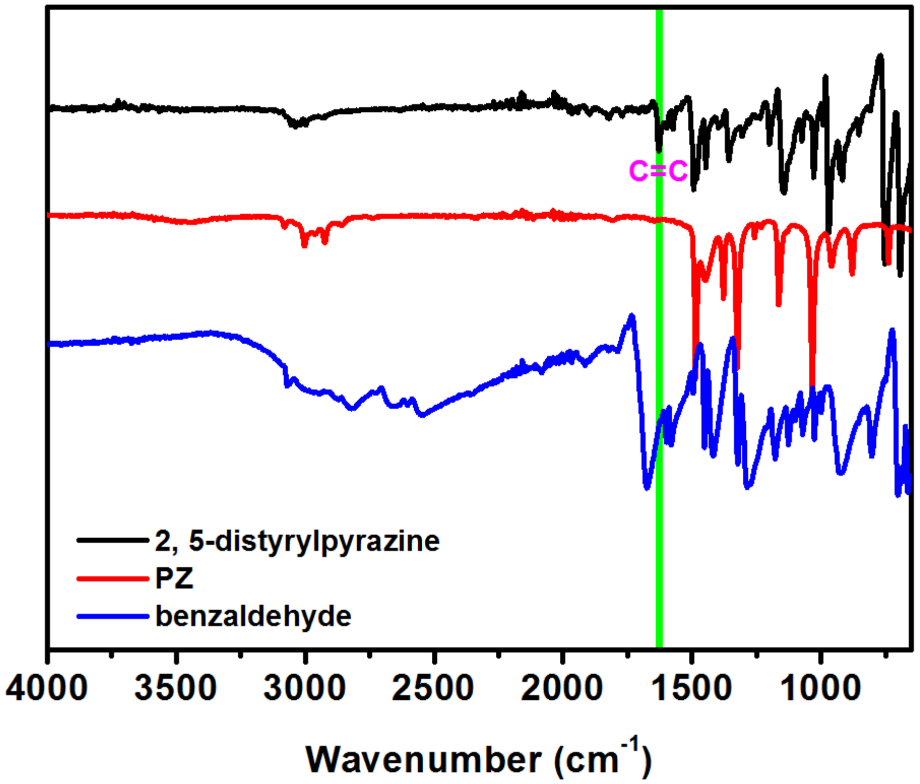

Supplementary Figure 3. FT-IR spectra of model molecule 2,5-distyrylpyrazine.

Supplementary Table 1. List of the reaction to fabricate olefin-linked COFs.

Entry	Compound	Reaction	Solvent	Catalyst	Ref.
1	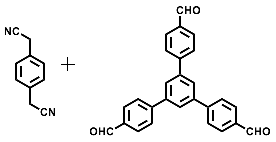	Knoevenagel condensation	1,2-dichlorobenzene	cesium carbonate	*Polym. Chem.* **2016**, *7,* 4176.
2	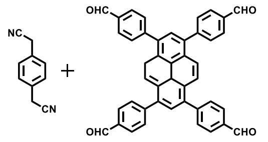	Knoevenagel condensation	mesitylene/ dioxane	NaOH	*Science* **2017**, *357,* 673.
3	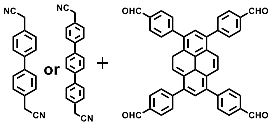	Knoevenagel condensation	1,4-dioxane /1,2-dichlorobenzene	KOH or TBAH	*Nat. Commun.* **2018**, *9,* 4143.
4	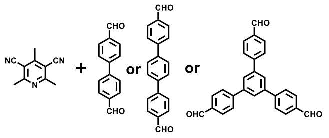	Knoevenagel condensation	DMF	piperidine	*Nat. Commun.* **2019**, *10,* 2467.
5	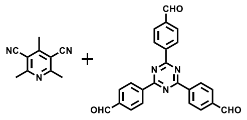	Knoevenagel condensation	DMF	piperidine	*Angew. Chem. Int. Ed.* **2019**, *131,* 12193.
6	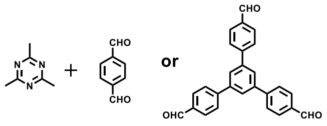	Knoevenagel condensation	n-butanol/o-dichlorobenzene	KOH	*J. Am. Chem. Soc.* **2019**, *141,* 14272.
7	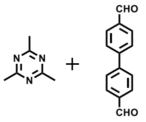	Aldol condensation	Mesitylene/ 1,4-dioxane/acetonitrile	trifluoroacetic acid	*J. Am. Chem. Soc.* **2019**, *141,* 6848.
8	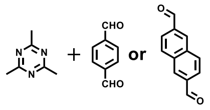	Aldol Condensation	Methanol/ Mesitylene	NaOH	*J. Am. Chem. Soc.* **2020**, *142,* 8862.
9	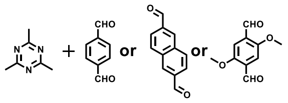	Aldol Condensation	dioxane/ethanol	NaOH	*Angew. Chem. Int. Ed.* **2019**, *58,* 13753.
10	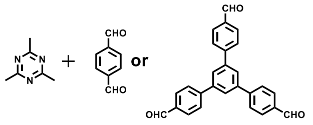	Aldol Condensation	Methanol/ Mesitylene	NaOH	*Angew. Chem. Int. Ed.* **2019**, *58,* 14865.
11	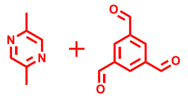	Aldol Condensation	No	benzoic anhydride	**This work**

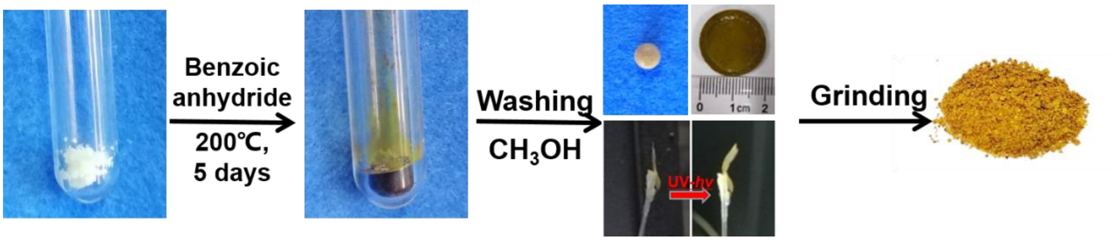

Supplementary Figure 4. The photographs showing the progress of polymerization of NKCOF-10.

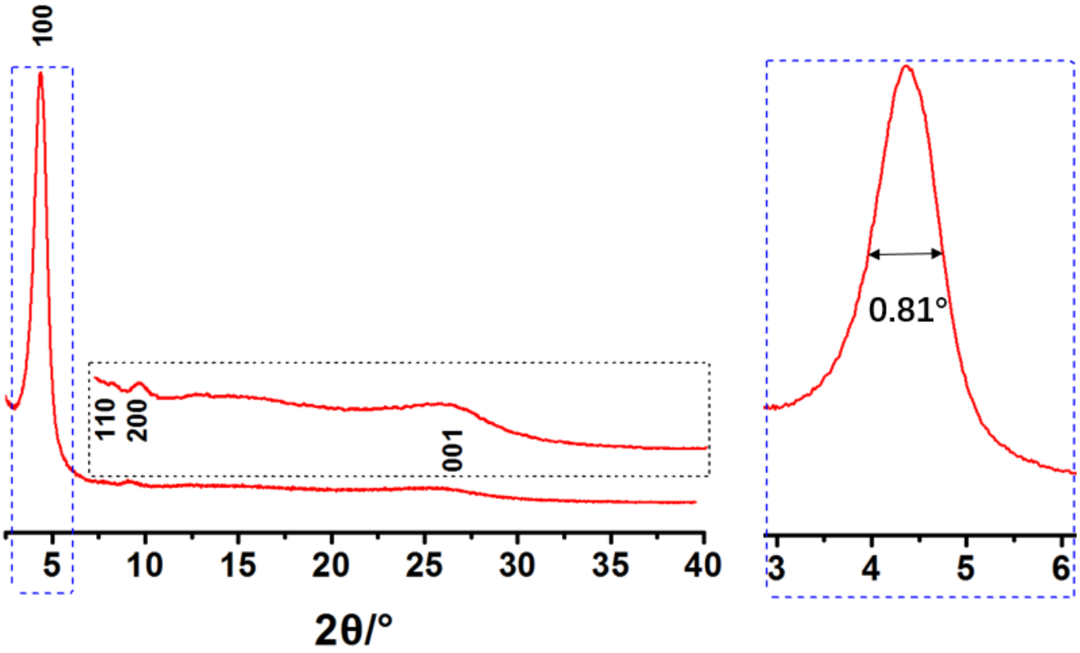

Supplementary Figure 5. PXRD patterns of NKCOF-10.

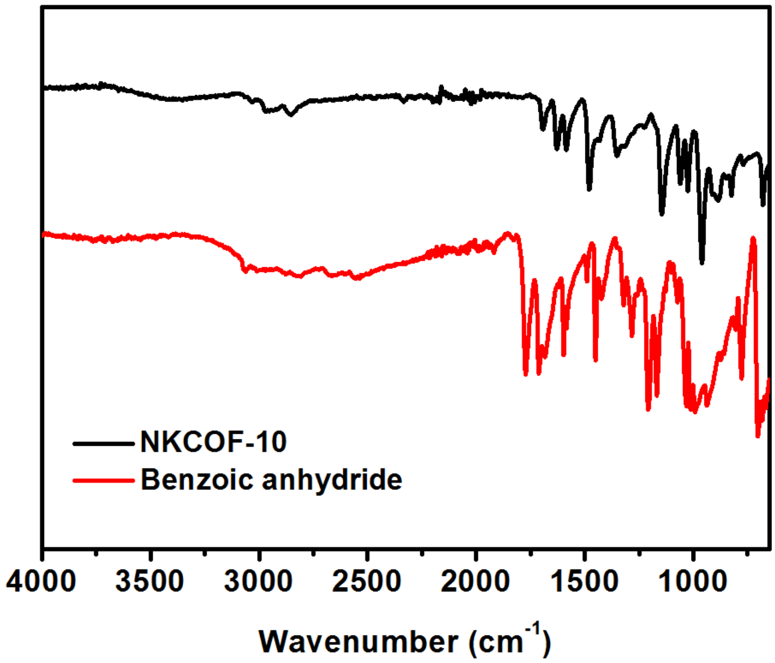

Supplementary Figure 6. FT-IR spectra of NKCOF-10 (red curve) and benzoic anhydride (black curve).

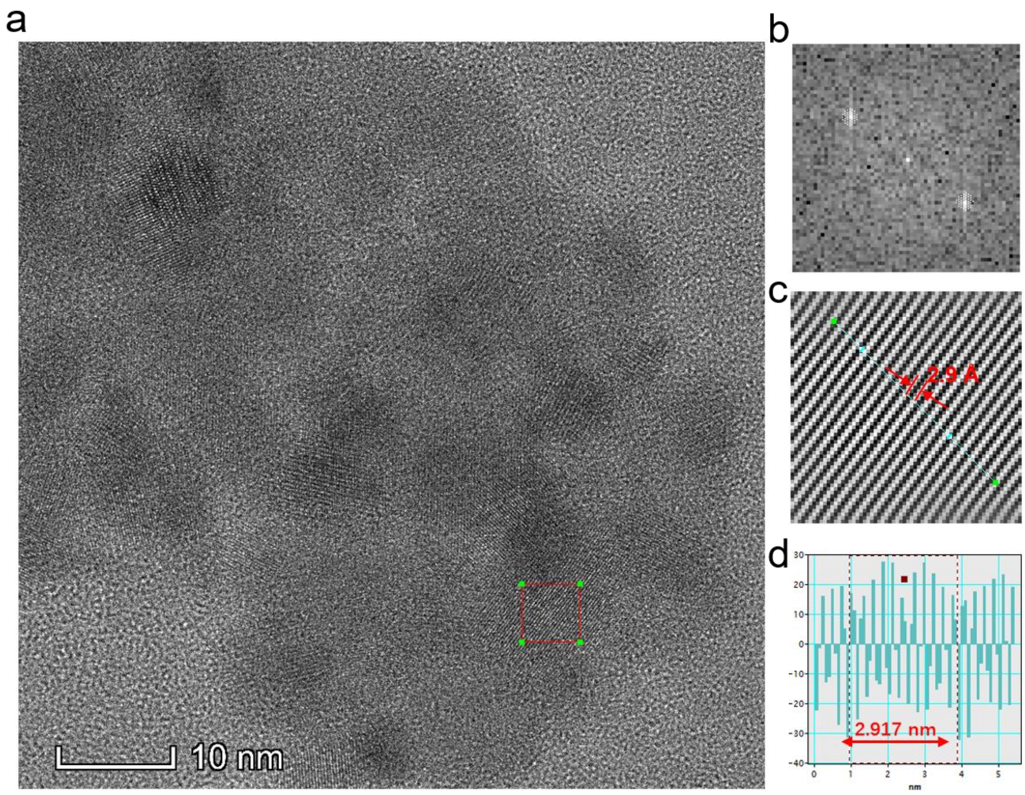

Supplementary Figure 7. **a** HRTEM image of NKCOF-10. **b** corresponding FFT of NKCOF-10. **c** HRTEM image of NKCOF-10 with layer to layer distance about 2.9 Å. **d** corresponding line profile along the indicated area in TEM image.

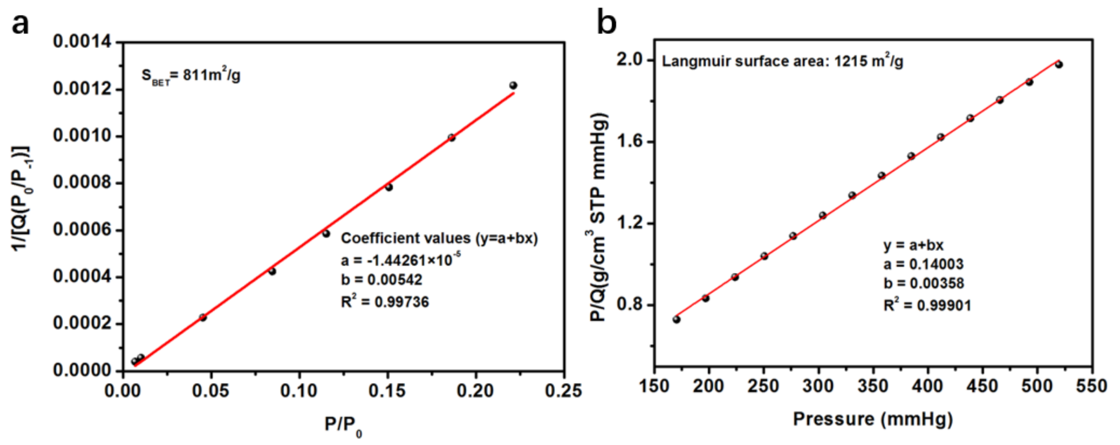

Supplementary Figure 8. BET plot (a) and Langmuir plot (b) from N_2 adsorption data at 77 K for NKCOF-10.

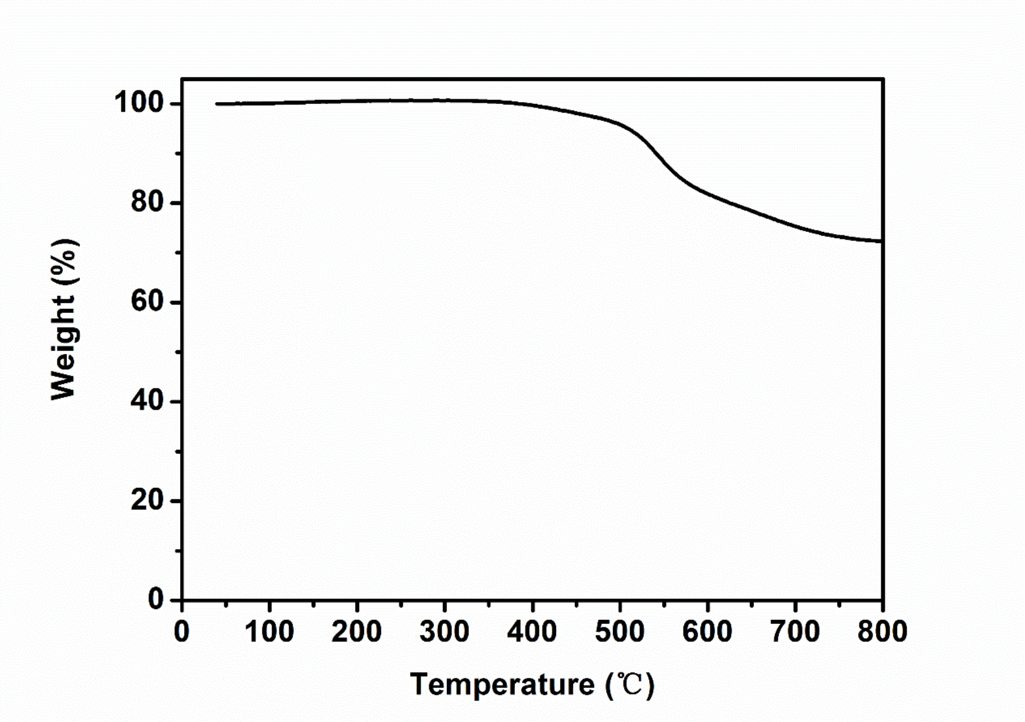

Supplementary Figure 9. TGA curve of NKCOF-10.

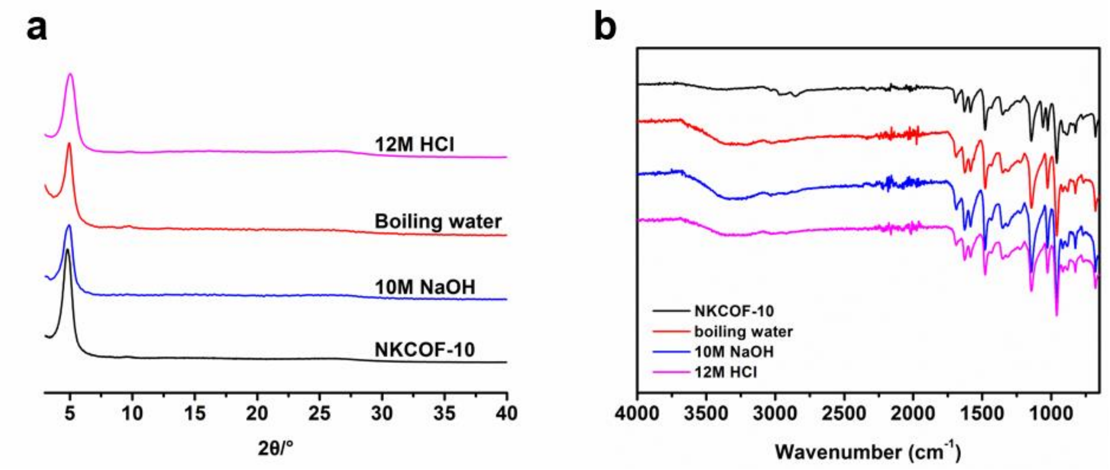

Supplementary Figure 10. a PXRD patterns of NKCOF-10 and **b** FT-IR spectra of NKCOF-10 after treatment in strong acid, base, and boiling water for 2 days.

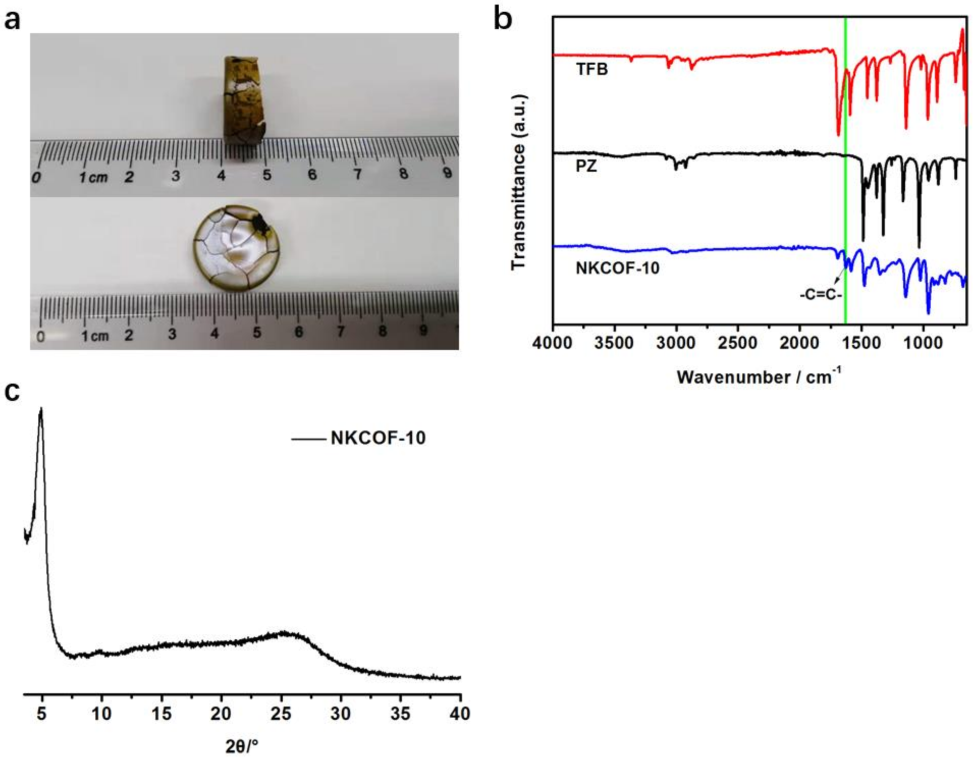

Supplementary Figure 11. Solvent-free synthesis of high crystalline COF in gram-scale. **a** Photographic images of the gram-scale NKCOF-10 sample. **b** FT-IR spectra of NKCOF-10 and corresponding monomers. **c** PXRD pattern of gram-scale NKCOF-10.

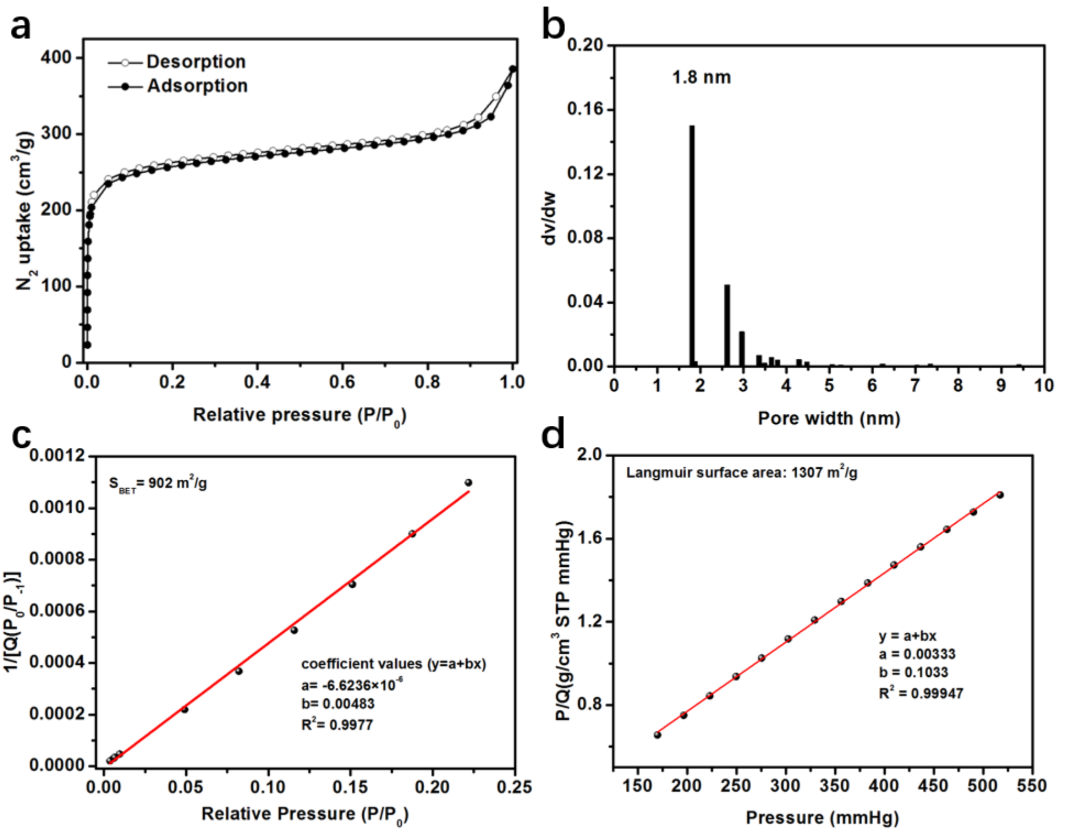

Supplementary Figure 12. a Nitrogen adsorption and desorption isotherms of the gram-scale NKCOF-10. **b** The pore size distribution. **c, d** BET and Langmuir plots from N_2 adsorption data at 77 K for NKCOF-10.

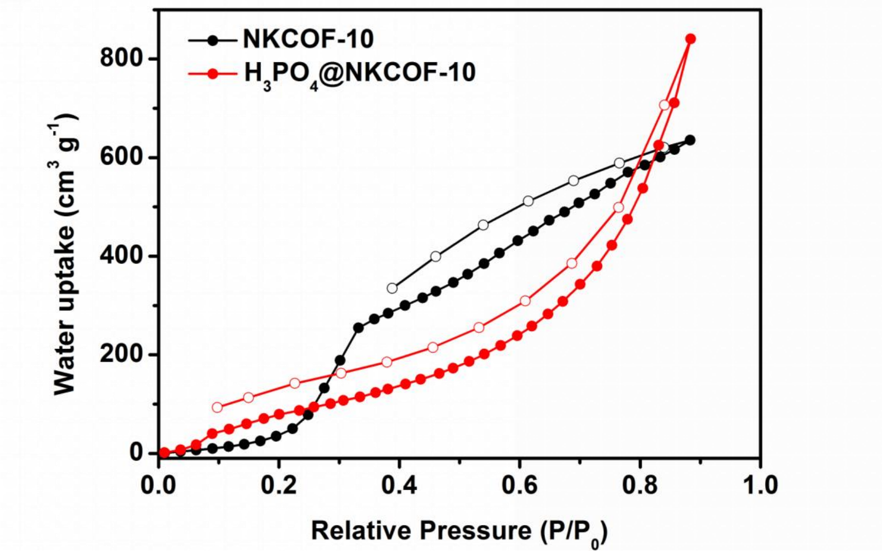

Supplementary Figure 13. Water vapor adsorption isotherm of NKCOF-10 (black) and H_3PO_4@NKCOF-10 (red) at 298 K, indicating the high hydrophilicity. Filled and open symbols correspond to adsorption and desorption processes, respectively.

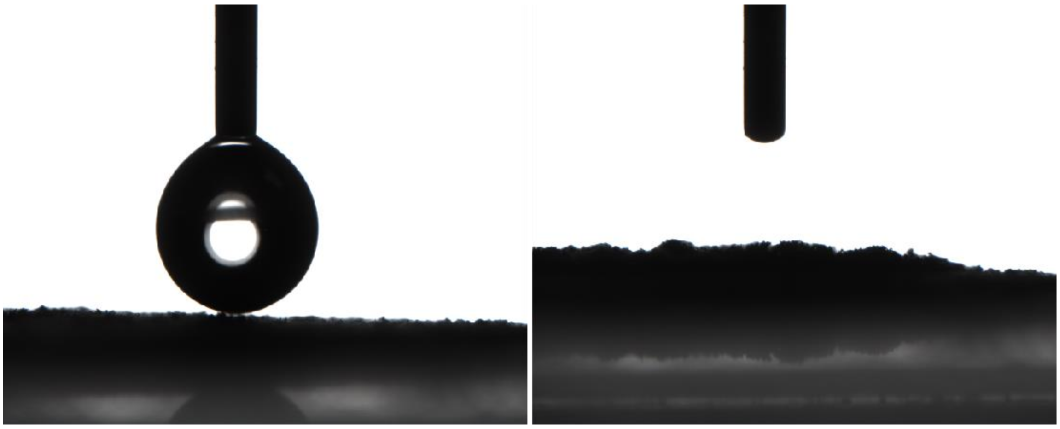

Supplementary Figure 14. Water contact angle measurement of NKCOF-10.

Supplementary Table 2. The proton conductivity of NKCOF-10 and H_3PO_4@NKCOF-10 at different temperatures under 90% RH and their corresponding activation energy.

Sample	Temperature (K)	Resistance (Ω)	Proton Conductivity (S cm^{-1})	Activation Energy (eV)
NKCOF-10<br>Diameter=1.3 cm<br>Thickness(d)=0.05 cm	303	28000	0.135×10^{-5}	0.40
	313	14000	0.27×10^{-5}	
	323	12000	0.315×10^{-5}	
	333	7400	0.51×10^{-5}	
	343	4500	0.84×10^{-5}	
	353	3500	1.08×10^{-5}	
H_3PO_4@NKCOF-10<br>Diameter=1.3 cm<br>Thickness(d)=0.028 cm	298	0.305	6.97×10^{-2}	0.06
	303	0.277	7.67×10^{-2}	
	313	0.267	7.96×10^{-2}	
	323	0.258	8.23×10^{-2}	
	333	0.253	8.40×10^{-2}	
	343	0.245	8.71×10^{-2}	
	353	0.239	9.04×10^{-2}	

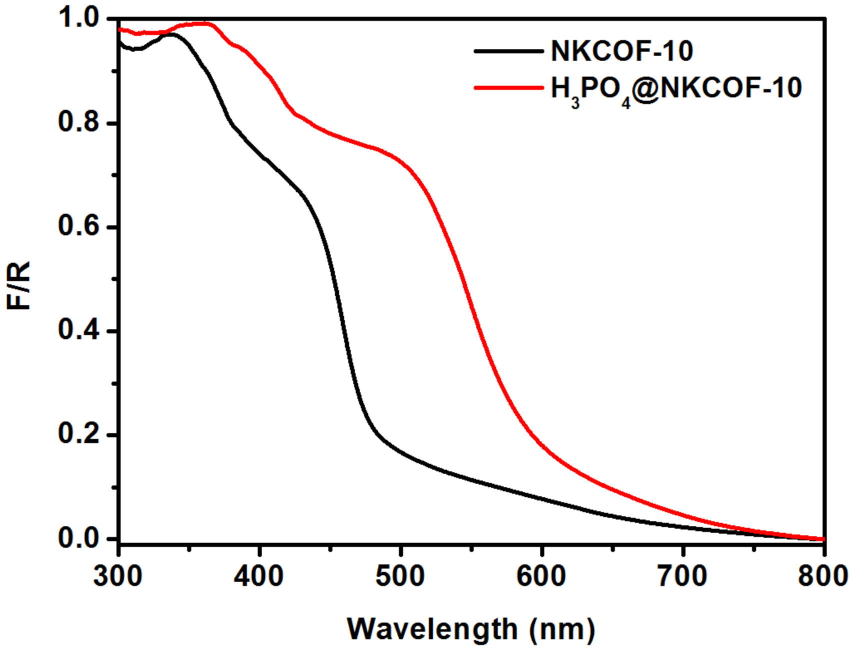

Supplementary Figure 15. Solid-state UV-Vis spectra of pristine NKCOF-10 (black) and H_3PO_4@NKCOF-10 (red).

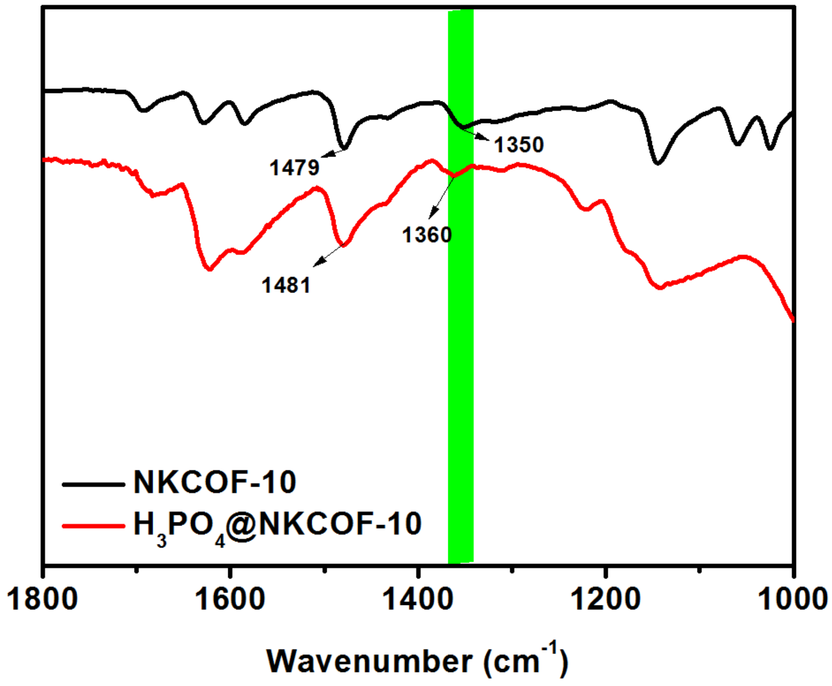

Supplementary Figure 16. FT-IR spectra of pristine NKCOF-10 (black) and H_3PO_4@NKCOF-10 (red).

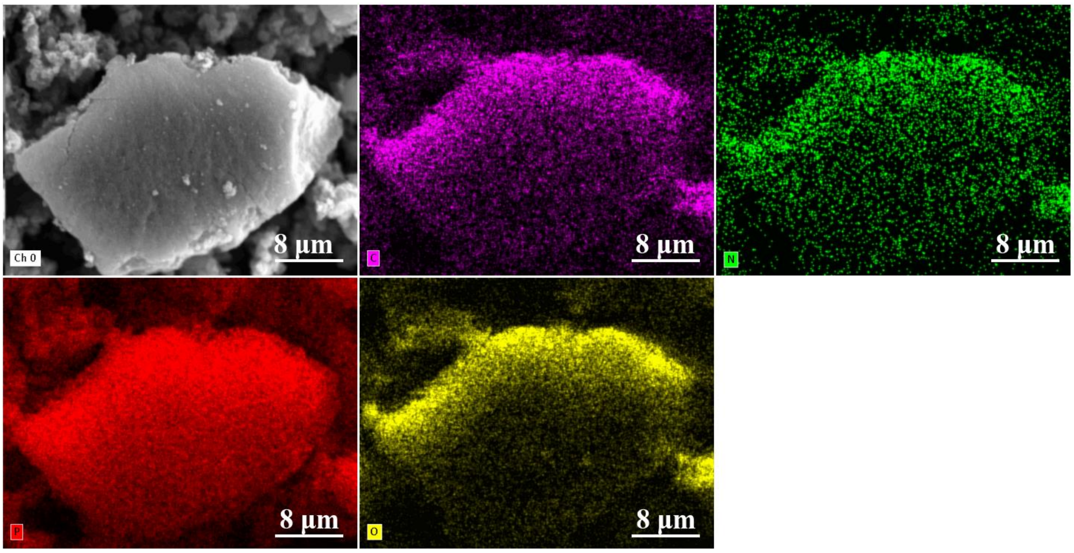

Supplementary Figure 17. Scanning electron microscopy (SEM) image and the corresponding mapping images of H_3PO_4@NKCOF-10.

Supplementary Table 3. List of the proton conductive materials based on COFs.

Sr. No	System	σ (S/cm)	RH (%)	T (K)	Ea (eV)	References
1	Nafion	~1 ×10^{-1}	98	353	0.22	*J. Polym. Sci., Part B: Polym. Phys.* **2011**, *49*, 1437.
2	**H_3PO_4@NKCOF-10**	**6.97×10^{-2}**	**90**	**298**	**0.06**	**This work**
3	**H_3PO_4@TPB-DMeTP-COF**	1.91×10^{-1}	0	433	0.34	*Nat. Commun.* **2020**, *11*, 1981.
4	**COF-F6-H**	4.2×10^{-2}	0	413	--	*J. Am. Chem. Soc,* **2020**, *142*, 14357.
5	**H_3PO_4@NKCOF-1**	1.13×10^{-1}	98	353	0.14	*Angew. Chem. Int. Ed.* **2020**, *59*, 3678.
	**H_3PO_4@NKCOF-2**	4.28×10^{-2}	98	353	0.24	
	**H_3PO_4@NKCOF-3**	1.12×10^{-2}	98	353	0.40	
	**H_3PO_4@NKCOF-4**	7.71×10^{-2}	98	353	0.08	
6	**PTSA@TpAzo COFM**	7.80×10^{-2}	95	353	0.11	*Angew. Chem. Int. Ed.* **2018,** *57*, 10894.
	**PTSA@TpBpy COFM**	6.20×10^{-2}	95	353	0.11	
	**PTSA@TpBD$(Me)_2$ COFM**	5.30×10^{-2}	95	353	0.23	
7	**NUS-9**	1.24×10^{-2}	98	298	--	*ACS Appl. Mater. Interfaces* **2016**, *8*, 18505.
	**NUS-10**	3.96×10^{-2}	98	298	--	
8	**BIP(COF)**	3.20×10^{-2}	95	368	0.31	*J. Am. Chem. Soc.* **2019,** *141*, 14950.
9	**CPOS-1**	1.00×10^{-2}	98	333	0.93	*Angew. Chem. Int. Ed.* **2018,** *57*, 1.
	**CPOS-2**	2.20×10^{-2}	98	333	0.61	
10	**RT-COF-1AcB**	5.25×10^{-4}	100	313	--	*J. Am. Chem. Soc.* **2017,** *139*, 10079.
	**RT-COF-1Ac**	1.07×10^{-4}	100	313	--	

	**LiCl@RT-COF-1**	6.45×10^{-3}	100	313	--	
11	**aza-COF-1H**	1.23×10^{-3}	97	323	0.29	*Chem. Meter.* **2019**, *31*, 891.
	**aza-COF-2H**	4.80×10^{-3}	97	323	0.45	
12	**EB-COF:PW12**	3.32×10^{-3}	97	298	0.24	*J. Am. Chem. Soc.* **2016,** *138*, 5897.
13	**im@TPB-DMTP-COF**	4.37×10^{-3}	0	403	0.38	*Nat. Mater.* **2016**, *15*, 722.
	**trz@TPB-DMTP-COF**	1.10×10^{-3}	0	403	0.21	
14	**PA@TpBpy-MC**	2.50×10^{-3}	0	393	0.11	*J. Mater. Chem. A* **2016**, *4*, 2682.
	**PA@TpBpy-ST**	1.98×10^{-3}	0	393	0.12	
15	**PA@Tp-Azo**	9.90×10^{-4}	98	333	0.11	*J. Am. Chem. Soc.* **2014,** *136*, 6570.
	**PA@Tp-Stb**	2.30×10^{-5}	98	333	0.14	
16	**phytic@TpPa-Py**	3.00×10^{-4}	0	393	0.10	*Chem. Meter.* **2016**, *28*, 1489.
	**phytic@TpPa-SO$_3$H**	7.50×10^{-5}	0	393	0.16	
17	**HOF-GS-10**	1.78×10^{-4}	60	303	0.13	*Angew. Chem. Int. Ed.* **2016,** *55*, 10667.
	**HOF-GS-11**	2.60×10^{-4}	60	303	0.48	

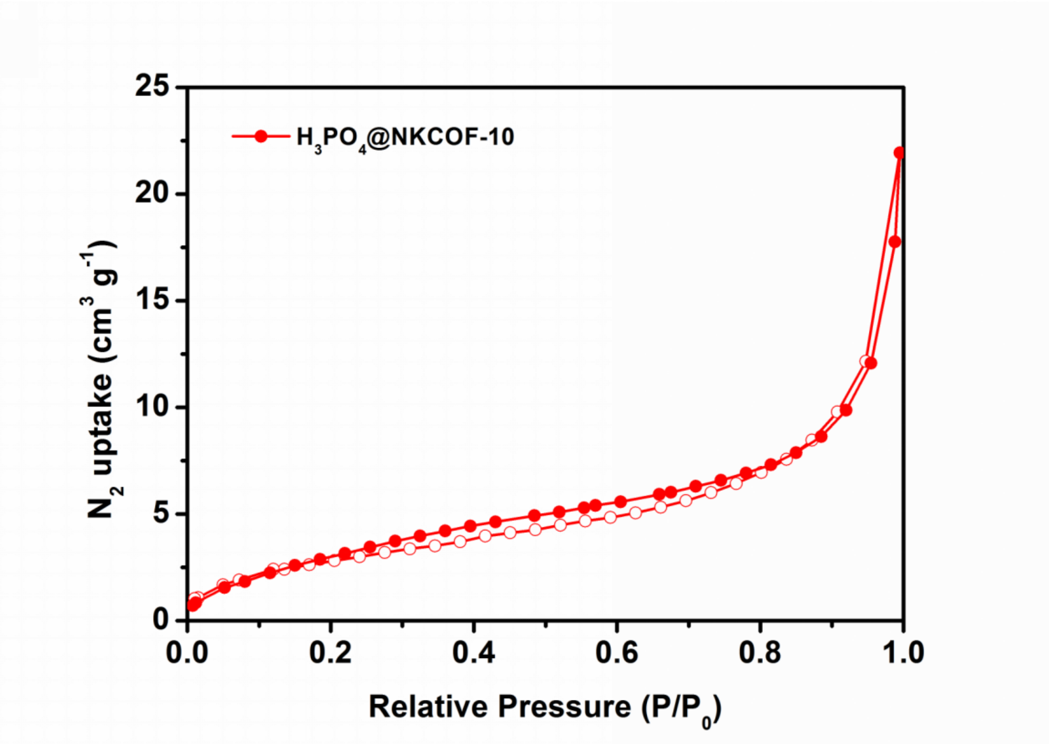

Supplementary Figure 18. Nitrogen adsorption and desorption of H_3PO_4@NKCOF-10.

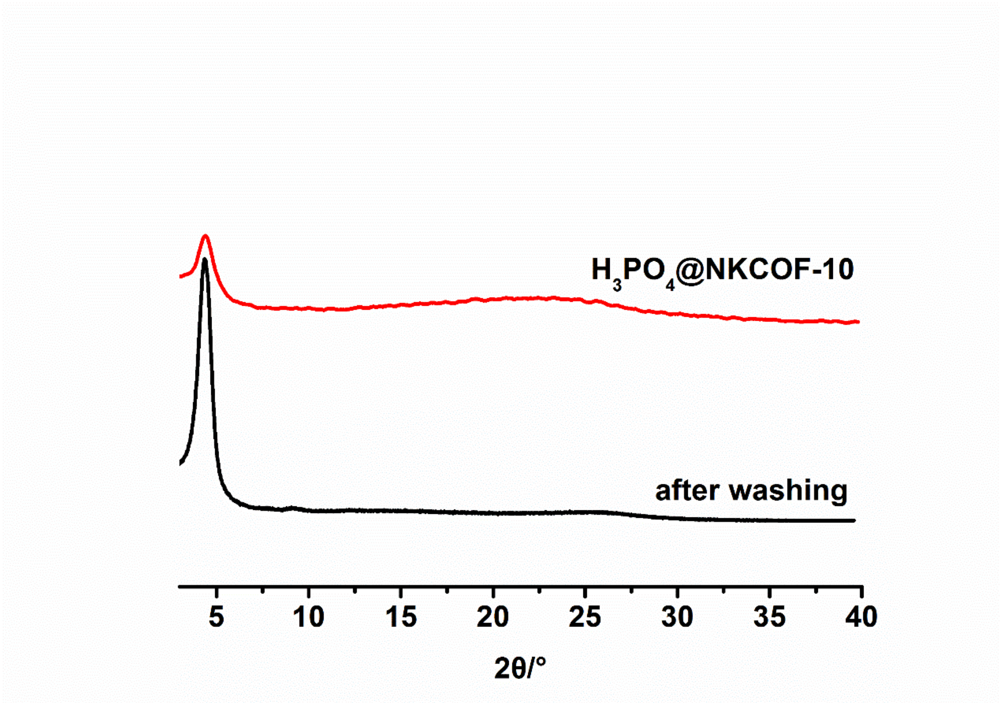

Supplementary Figure 19. PXRD patterns of H_3PO_4@NKCOF-10 and after washing sample with saturated aqueous $NaHCO_3$ solution, water, and alcohol.

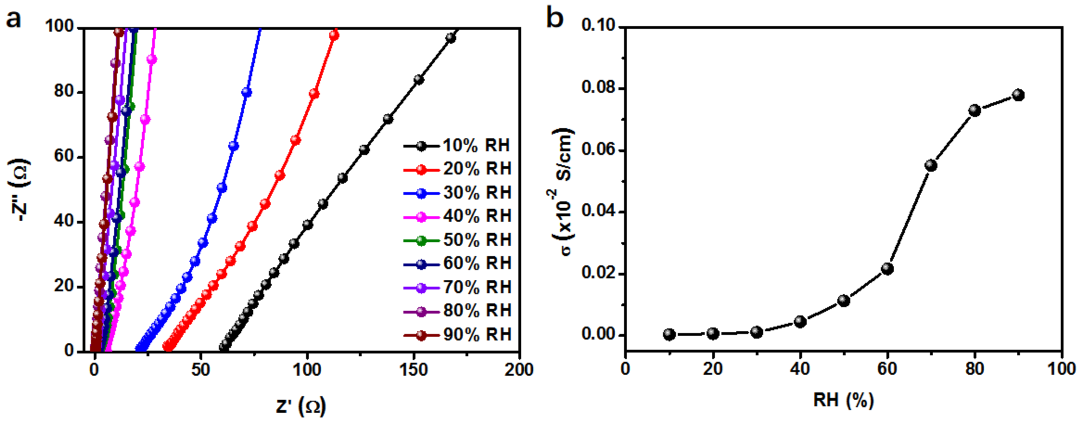

Supplementary Figure 20. a Nyquist plots of H_3PO_4@NKCOF-10 measured at 323 K under different relative humidity. **b** Proton conductivity of H_3PO_4@NKCOF-10 under different relative humidity when fixing temperature at 323 K.

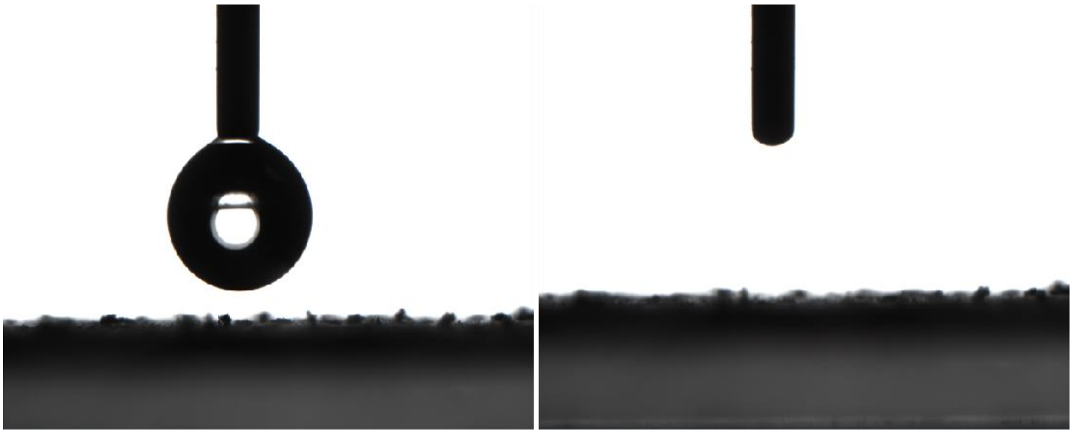

Supplementary Figure 21. Hydrophobic angle measurement of H_3PO_4@NKCOF-10.

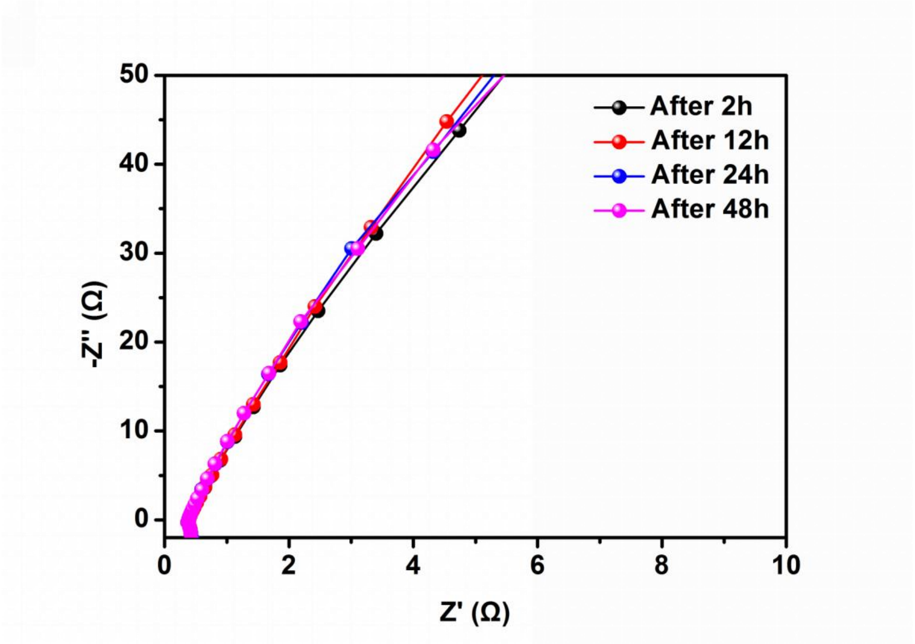

Supplementary Figure 22. Nyquist plots of H_3PO_4@NKCOF-10 measured at 333 K under 90% RH for 48 consecutive hours in a constant temperature and humidity chamber.

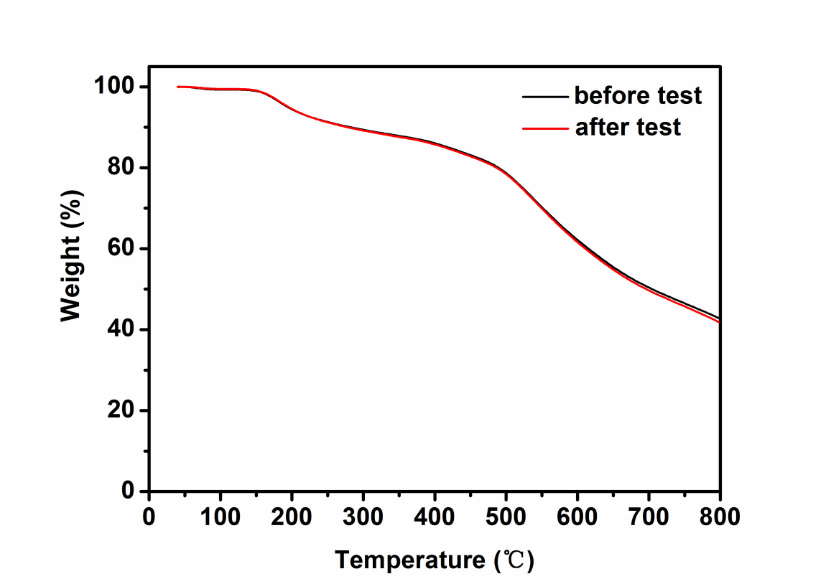

Supplementary Figure 23. TGA curves of H_3PO_4@NKCOF-10 before and after EIS test. TGA data revealed that the loading of phosphoric acid kept the same values before and after EIS measurement, further indicating no leaching of phosphoric acid in NKCOFs.

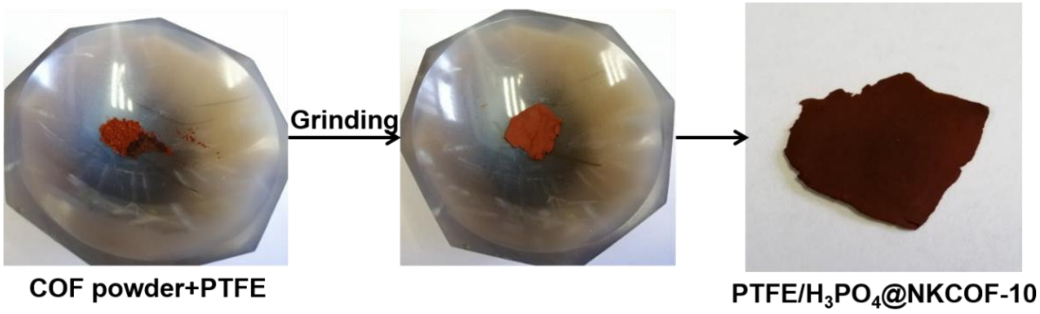

Supplementary Figure 24. The photographs showing the progress of H_3PO_4@NKCOF-10 membranes**.**

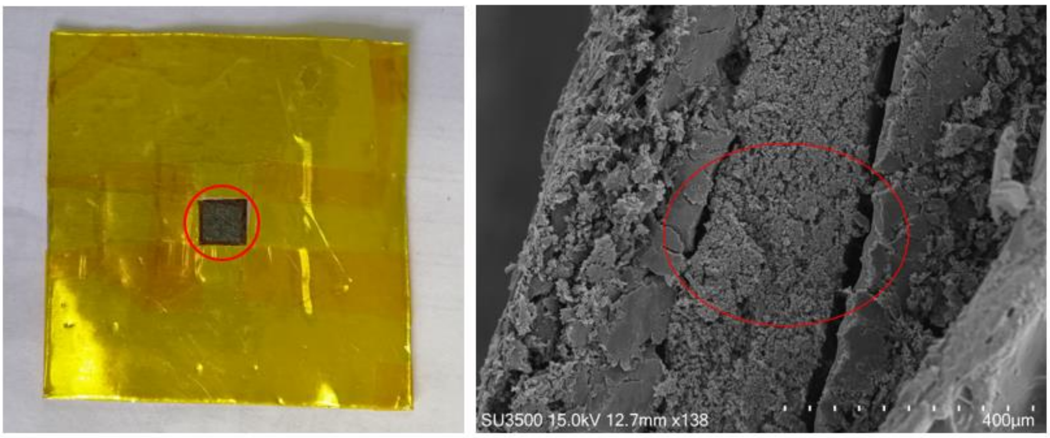

Supplementary Figure 25. Optical image and cross-section SEM image of solid electrolyte membranes based on H_3PO_4@NKCOF-10.

Supplementary References

1. Hogue, R. W. et al. Self-assembly of cyclohelicate [M_3L_3] triangles over [M_4L_4] squares, despite near-linear bis-terdentate L and octahedral M. *Chem. Eur. J.* **23**, 14193-14199 (2017).
